# Biobanks: understanding chronic diseases in their complexity

**DOI:** 10.1186/1753-6561-7-S5-O15

**Published:** 2013-08-30

**Authors:** N Probst-Hensch

**Affiliations:** 1 Department of Epidemiology & Public Health, Swiss TPH, Switzerland

## 

The concept of biobanks can well be explained through the example of SAPALDIA cohort study which involves multicentric collection and central storage of blood, plasma, serum, buffy coat and DNA extracts. Personalized medicine through genomic sequencing and genetic risk profiling is very much a possibility with the publication of genome-wide associations. Even though access to complete genome sequencing is technically feasible the clinical utility and relevance of DNA sequencing is still very poorly understood and it should not be assumed that research will provide answers quickly. Genomics helps in the study of gene-environment interactions thus contribution to insights in genetic epidemiology. But genetic testing can also be a motivating factor for behavioural change.

Limitations in environmental epidemiology of chronic diseases has left several gaps in understanding the causality of risk factors, etiological pathways and shared pathways. The environment and lifestyle varies over time, exposures are difficult to measure and susceptibilities have been ignored in the past. Biobanking and genomic studies are important in understanding the pathways by which risk factors and behaviours result in chronic diseases in a particular environmental context.

The exposome concept refers to the exposures of an individual over a lifetime and the relation of these exposures to health. The exposome changes throughout life as our bodies, diets and lifestyles change. Measurement of exposures and their effects are important in describing the exposome and understanding the interaction between environmental exposures and genetic and epigenetic mechanisms. The use of biomarkers to determine exposures, effect of exposures, disease progression and susceptibility factors are common to the “omics” technologies of genomics, transcriptomics, proteomics and metabonomics. A blood or urine sample taken from an individual could provide a snapshot of what that person has been exposed to. The metabolic profile thus potentially reflects the interactions between the exposome and genome with the ability to provide insights into causal pathways of complex, chronic diseases.

Metabonomics refers to the study of metabolic responses to environmental changes, drugs, genetic modifications and it involves measurement of small molecular weight compounds which may act as regulatory signals in the biological system. Examples of metabonomic studies include the INTERMAP study which is a multi-centric investigation into the role of dietary factors in development of hypertension in adults. Study populations differ by ethnicity, diet, diet-related major cardiovascular risk factors and prevalence of hypertension/coronary heart disease/stroke. Another study involving identification of urinary metabolites suggests well differentiated metabolic phenotypes. Most discriminatory metabolites are of dietary origin or gut microbial co-metabolites. Candidate biomarkers emerging from other studies include formate for blood pressure, proline-betaine as a marker for citrus consumption and C-Reactive protein as a marker in the pathway of coronary heart disease. In addition several genetically determined metabolic phenotypes are also being identified. The expected benefits from studying exposomes and biomarkers include decreased exposure misclassification , identification of early disease markers , assigning causality, identification of determinants of susceptibility and the understanding of biological mechanisms . Complex chronic diseases can be studied through biomarkers of susceptibility, exposure and disease. Understanding the differential burden of chronic diseases and risk factors across different ethnic, cultural and geographic settings through biomarkers can improve the causal understanding. Establishing mega cohorts and international cohorts can help better understanding of lifestyle, environment, biomarkers and diseases through proper networking. They provide important policy inputs, maintain and strengthen competitive research, help scientific and technological advancement in addition to funding and job opportunities.

